# Carcinome basocellulaire chez un albinos congolais (République Démocratique du Congo): à propos d'une observation

**DOI:** 10.11604/pamj.2015.20.274.6356

**Published:** 2015-03-20

**Authors:** David Kakez Nday, Léon Kabamba Ngombe, Jimmy Ngoie Fundi, Tony Kayembe Kitenge, Luboya Numbi

**Affiliations:** 1 Zone de Santé de Dilolo, Hôpital General de Dilolo, Dilolo, République Démocratique du Congo; 2 Université de Kamina, Faculté de Médecine, Département de Santé Publique, Unité de Toxicologie, République Démocratique du Congo; 3 Université de Lubumbashi, Faculté de Médecine, Département de Santé Publique, Unité de Toxicologie, Lubumbashi, République Démocratique du Congo; 4 Zone de Santé de Kolwezi, Hôpital General de Kolwezi, Kolwezi, République Démocratique du Congo; 5 Université de Lubumbashi, Faculté de Médecine, Département de Pédiatrie, République Démocratique du Congo

**Keywords:** Carcinome basocellulaire, sujet noir, congolais, albinos, vendeur, basal Cell Carcinoma, Black African, Congolese, albinos, seller

## Abstract

Les auteurs rapportent un cas d'un carcinome basocellulaire non décris dans la littérature de notre pays chez un adulte jeune congolais âgé de 25 ans, de sexe masculin présentant une récidive probable de la tumeur. Cette observation permet de décrire le carcinome basocellulaire chez un sujet noir albinos, et de souligner les particularités thérapeutiques.

## Introduction

Le carcinome basocellulaire (CBC) est la tumeur maligne la plus fréquente chez les êtres humains [1–3]. Il est fréquemment présent dans les régions qui sont exposées chroniquement au soleil [3, 4]. En général, le CBC est une tumeur qui se développe lentement [2]. Cette tumeur maligne cutanée est plus fréquente chez les caucasiens, mais, elle est rare chez la population noire. Le CBC représente 2% à 8% des cancers cutanés chez les noirs africains [5] et 12% à 35% chez les noirs américains [6]. Dans notre milieu, il n'y a pas des données relatives à cette tumeur. Ce travail a pour but de décrire le carcinome basocellulaire chez un sujet noir albinos, et de souligner les particularités thérapeutiques.

## Patient et observation

**Figure 1:**
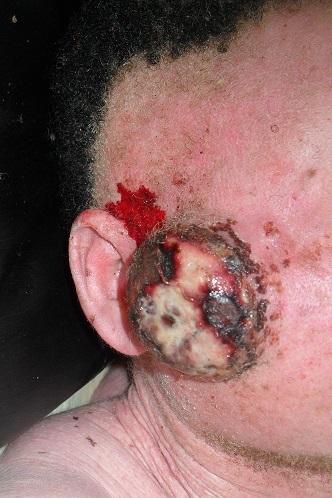
La masse récidive au même endroit mais du coté droite

**Figure 2:**
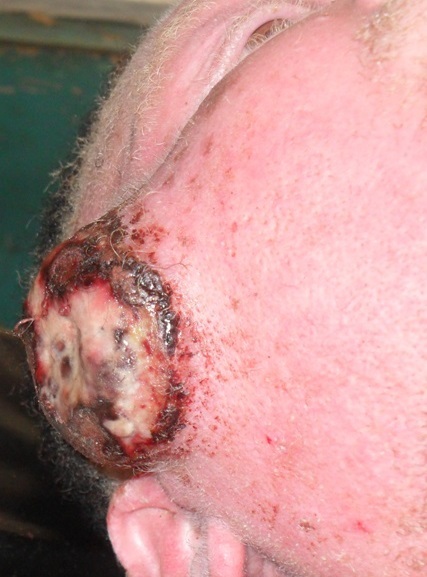
Une tuméfaction d'allure kystique dans la région temporale droite

**Figure 3:**
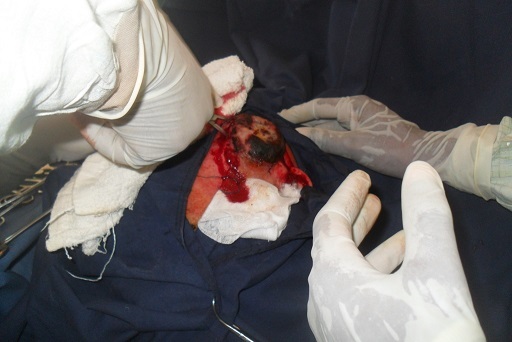
Une exérèse large

**Figure 4:**
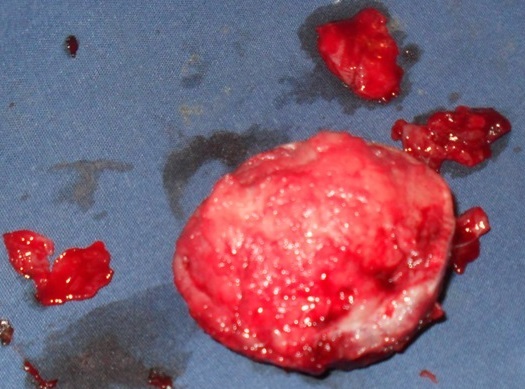
La tuméfaction d'allure kystique

Il s´agit d´un homme de 25 ans, de race noire, albinos, vendeur ambulant des disques musicaux qui avait comme plainte actuelle une tuméfaction faciale récidivante. Dans ses antécédents, on retrouvait une notion d´exérèse d´une masse géante dans la région temporale gauche sans examen histologique effectué. Une année après cette intervention chirurgicale, la masse récidive au même endroit mais du coté droite (Figure 1). Ce qui pousse le patient à venir consulté une fois de plus. L´examen physique retrouvait une tuméfaction d´allure kystique dans la région temporale droite (Figure 2), mesurant 13 mm sur le plus grand axe, surmontée de quatre petites ulcérations et le reste de l´examen physique est sans particularité. Le traitement avait consisté en une exérèse large de la tuméfaction d´allure kystique (Figure 3). L´endoxan a été donné au patient avant et après l´intervention. L´examen anatomo-pathologique de la pièce opératoire a révélé un carcinome basocellulaire chez un albinos de race noire (Figure 4).

## Discussion

Les cancers de la peau (carcinomes baso-cellulaires et spino-cellulaires) sont plus fréquents au sein de la population d´origine caucasienne. Le CBC représente 2% à 8% des cancers cutanés chez les noirs africains [5] et 12% à 35% chez les noirs américains [6]. Cependant, il n´existe pas de travaux approfondis et définitifs sur l´incidence et/ou la prévalence des cancers cutanés au sein des populations africaines. Néanmoins, les études menées en milieu hospitalier africain révèlent la relative rareté de ces tumeurs malignes comparativement aux pays à fort peuplement caucasien [7]. En effet, le sex-ratio est variable surtout dans les études des patients des races noires [6] mais certaines études ont montré une incidence quasi égale entre les deux sexes [5]. Notre observation ne pourra pas montre le sexe ayant une incidence supérieure à l´autre. Selon la littérature, le CBC apparait le plus fréquemment à l´âge adulte, en particulier à partir de la 5eme décennie de vie [8]. Par contre, Ademiluyi et Ijaduola rapportent que l´africain albinos développe le BCB dès le bas âge par rapport au noir africain [9]. Ceci pourrait être valable pour notre observation.

Cliniquement, la tumeur chez notre patient était localisé au niveau de la face avec l´absence des ganglions cervicaux et sus claviculaires. Ce constat est conforme aux données de la littérature qui localisent le CBC dans deux tiers des cas au niveau de la tête et du cou car ces régions sont exposées chroniquement au soleil [3, 4]. Par ailleurs, l´absence des adénopathies est une preuve illustrant que le CBC est parmi les cancers de la peau n´ayant pas la capacité de donner des métastases, bien que la littérature rapporte quelques cas des métastases [10].

Il est connu que la forte pigmentation cutanée des africains les protégerait contre les carcinomes cutanés. Ainsi, l´incidence du CBC semble directement corrélée avec le degré de pigmentation de la peau. De ce fait, l´albinisme constituerait dans ce contexte un facteur de risque [11]. D´autres facteurs sont notamment: les rayons ultraviolets (UV) entre 290 et 400nm, les rayonnements ionisants, les hydrocarbures aromatiques polycycliques, l´arsenic, les cicatrices des brulures et certains syndromes génétiques (Xeroderma pigmentosum, neavomatose basocellulaire, epidermodysplasie verruciforme) sont également impliqués comme favorisant la survenue du carcinome basocellulaire [12]. En ce qui nous concerne, l´albinisme, le manque d´utilisation des matériels de protection (crème antisolaire, port du chapeau, les habits noirs à manche longue), et l´exposition solaire répétée du fait de sa profession sont fortement incriminés dans la genèse du CBC chez notre patient. De nos jours, il est connu que le CBC qui n´est pas traité complètement peut récidiver et par conséquent toutes les régions traitées doivent être surveillées après traitement [13, 14]. Malheureusement, il est difficile pour nous de parler d´un CBC récidivant car il n´y avait pas de biopsie faite lors de la première exérèse et que la masse devrait récidiver en principe dans la même région (à gauche). Curieusement, une année après, la masse réapparait dans la région opposée (à droite) chez notre patient qui est un albinos avec une biopsie révélant un CBC. Ce phénomène rare dans la littérature dans notre pays attire notre attention et mérite d´être mentionné.

## Conclusion

Le carcinome basocellulaire (CBC) est la tumeur maligne la plus fréquente chez les êtres humains. Les régions exposées fréquemment au soleil sont les plus atteintes. La prévention s'avère importante car elle est basée sur l'information de la population et l'usage des matériels de protection (crème antisolaire, port du chapeau, les habits noirs à manche longue). L'efficacité de la prise en charge réside dans la multidisciplinarité de l’équipe à savoir: médecin généraliste, dermatologue, chirurgiens plasticiens, anatomopathologistes et cancérologues.
